# Activated Human Umbilical Cord Blood Platelet-Rich Plasma Enhances the Beneficial Effects of Human Umbilical Cord Mesenchymal Stem Cells in Chemotherapy-Induced POF Rats

**DOI:** 10.1155/2021/8293699

**Published:** 2021-10-25

**Authors:** Jiawei Wang, Yunxiao Zhao, Fengque Zheng, Nana Ma, Rongyan Qin, Weili Qin, Bo Liu, Aiping Qin

**Affiliations:** Center of Reproductive Medicine, The First Affiliated Hospital of Guangxi Medical University, Nanning 530000, China

## Abstract

Saving the ovarian function of premature ovarian failure (POF) patients undergoing chemotherapy is an important problem in the field of reproductive medicine. At present, human umbilical cord mesenchymal stem cells (HucMSCs) have been used in the treatment of POF, but the effect is still not optimal. The purpose of this study was to determine whether human umbilical cord blood platelet-rich plasma (ucPRP) enhances the beneficial effects of HucMSCs in the treatment of POF. First, we observed the effects of changes in the biological activity of ucPRP on HucMSCs in vitro. Subsequently, we tracked the distribution and function of the HucMSCs in POF rats, and the rats' estrus cycle and serum sex hormones, follicular development, ovarian angiogenesis, ovarian granulosa cell proliferation, and apoptosis were assessed. The results of the study showed that the addition of ucPRP in vitro accelerates proliferation and reduces apoptosis of the HucMSCs while upregulating the stemness gene of the HucMSCs. The combined transplantation of HucMSCs and ucPRP resulted in more stem cells being retained in the ovaries of POF rats, the estrus cycle of the POF rats being restored, the levels of serum E2, AMH, and FSH improving, and damaged follicles beginning to grow. Finally, we confirmed that the potential mechanism of the combination of HucMSCs and ucPRP to rescue the ovarian function of POF rats is to promote ovarian angiogenesis and to promote the proliferation and reduce the apoptosis of ovarian granulosa cells. The upregulation of AMH and FHSR expression and the downregulation of caspase-3 expression in granulosa cells are potential mechanisms for the recovery of ovarian function. Our research results suggest that the combined application of HucMSCs and ucPRP is a safe and efficient transplantation program for the treatment of POF, thus providing a reliable experimental basis for the clinical application of stem cell therapy in POF.

## 1. Introduction

Premature ovarian failure (POF) is clinically defined as decreased ovarian function in young women and mainly manifests as abnormal menstruation, abnormal increase in FSH, decreased estrogen fluctuations, and even infertility [1]. POF is usually caused by factors such as genetics, immunity, radiotherapy, and chemotherapy. Approximately 5% of cancer patients requiring chemotherapy are young women [2]. Restoring the ovarian function in young women undergoing chemotherapy with an associated loss of fertility is a problem that urgently needs to be solved. There are many methods for the treatment of POF: hormone replacement therapy (HRT), immunomodulation therapy, and ovarian tissue cryopreservation. However, these treatments have certain limitations, including increased risk of breast or ovarian cancer, fatal allergic reactions, complicated surgical procedures, and inability to promote damaged ovarian tissue regeneration [3–5].

Stem cell therapy, as the most promising treatment to promote tissue damage repair, has been used for the regeneration of various tissues. Mesenchymal stem cells (MSCs) belong to the family of multipotent stem cells, which have the characteristics of stem cells: self-renewal, homing, and directed differentiation abilities [6–8]. Among these cells, human umbilical cord mesenchymal stem cells (HucMSCs) are derived from the umbilical cord of fetal delivery appendages, with a wide range of sources, less ethical controversy, and a low immunogenicity, and are considered the best choice for stem cell therapy [9, 10]. Recently, two cases reported the exciting results that HucMSCs partially improved the ovarian function of POF rats [11, 12]. However, the low survival rate and uncertain efficacy of HucMSCs after transplantation still limit their clinical application. Improving the efficacy of HucMSCs has become the greatest challenge for stem cell transplantation treatment.

Platelet-rich plasma (PRP) is mainly composed of growth factors such as platelet-derived growth factor (PDGF), fibroblast growth factor (FGF), epidermal growth factor (EGF), and transforming growth factor *β*-1 (TGF-*β*1). It has been shown to play a powerful role in the repair of various types of tissue damage, including osteoarthritis repair, retinal repair, kidney damage repair, and skin damage repair [13–16]. The latest research shows that autologous PRP transplantation improves ovarian endocrine function and gives some POF patients a chance of pregnancy [17, 18]. Usually, platelet-rich plasma is separated from venous blood. Studies have shown that human umbilical cord blood platelet-rich plasma (ucPRP) derived from the umbilical cord has more growth factors than platelet-rich plasma derived from peripheral blood, such as PDGF and FGF. These growth factors play a decisive role in the proliferation and differentiation of MSCs [19, 20].

Both HucMSCs and ucPRP have demonstrated powerful tissue repair functions in POF, and we hypothesize that their combined use can accelerate the recovery of damaged ovaries. In our in vivo and in vitro studies, we observed the effect of ucPRP on the survival of HucMSCs. The surviving HucMSCs saved the function of the damaged ovaries [21]. We initially explored the beneficial effects and potential mechanisms of the combined application of HucMSCs and ucPRP. It has been proven that the combined transplantation of HucMSCs and ucPRP results in a more effective clinical treatment plan for POF.

## 2. Materials and Methods

### 2.1. HucMSC Isolation and Culture

We abided by the requirements of the ethics committee of the First Affiliated Hospital of Guangxi Medical University. With the permission of the patients, we obtained donated human umbilical cord tissue from patients who gave birth at the age of 25-30. The simple process used is as follows: we placed the collected umbilical cord in PBS containing 10% antibiotics (Gibco, USA) on a sterile operating table, removed the blood and mucus, cut the cord into small pieces of tissue (2 mm), and adhered it on the bottom of the petri dish; the culture dish was inverted in the incubator for 2 hours, and the tissue block was allowed to dry, to which alpha-MEM containing 10% FBS (Gibco, USA) was added, after which it was placed in the incubator to continue culturing until the HucMSCs crawled out of the tissue block. After the cells had reached 90% confluency, they were cultured for 5 generations for the subsequent experiments [22].

### 2.2. HucMSC Identification and Differentiation Potential

According to the requirements of the International Society for Cellular Therapy (ISCT) position paper, we identified purified HucMSCs by flow cytometry (BD Biosciences, USA) and a Human MSC Analysis Kit (562245, BD, USA). To identify the trilineage potential of HucMSCs, 2 × 10^4^ HucMSCs were seeded in a 6-well plate, and the osteogenic differentiation medium (HUXUC-90021, Cyagen, China) was replaced after the cells were 70% confluent, and the medium was refreshed every 3 days. After 21 days of induction, the cells were observed under a microscope by staining with an Alizarin Red staining solution. According to the instructions of the adipogenic kit (HUXUC-90031, Cyagen, China), HucMSCs were cultured in a 6-well plate to a confluency of 100%, and then, medium A solution was added for incubation for 3 days, followed by replacement with the B solution for 1 day. After four cycles, the B solution was replaced, followed by incubation for 5 days. The cells were stained with an Oil Red O staining solution and observed under a microscope. In accordance with the instructions of the chondrogenic differentiation kit (HUXUC-90041, Cyagen, China), HucMSCs were incubated in chondrogenic differentiation medium, and the medium was refreshed every 3 days. After the cultivation for 21 days, the Alcian blue-stained cells were observed under a microscope. Undifferentiated HucMSCs were stained with Alizarin Red, Oil Red O, and Alcian blue as control staining, respectively; the cells were observed under a microscope (Olympus BX53F, Japan).

### 2.3. Preparation of ucPRP

With the permission of the ethics committee and the patient's informed consent, the cord blood of healthy women aged 25-30 was collected in a test tube with sodium citrate (L14068, ShoeBio, China). According to the two-part centrifugation method, the samples were first centrifuged at 250 × *g*. After 10 minutes, the supernatant was removed and centrifuged again at 1000 × *g* for 10 minutes to remove the upper layer. The remaining 0.4 mL was regarded as ucPRP, and the platelets were resuspended to a concentration of 3 × 10^6^/*μ*L. Then, 20% CaCl_2_ (Sigma-Aldrich, USA) and 1000 U/mL thrombin (T8021, Solarbio, China) were added to the platelet mixture, which was incubated at 37°C for 1 h and then centrifuged at 12000 × *g* for 30 min to completely precipitate the jelly containing the cell debris and the fibrin clots. The collected supernatant was filtered with a 0.22 nm mesh and stored at -80°C to avoid repeated freezing and thawing [23].

### 2.4. EdU Detection

An EdU assay was performed to indicate the proliferation-promoting effect of ucPRP on HucMSCs. EdU is a thymidine analog that can penetrate replicating DNA molecules during cell proliferation. We set up a 10% FBS control group (*n* = 3) and an intervention group containing 10% ucPRP (*n* = 3). HucMSCs (4 × 10^3^) were inoculated into a 96-well plate, incubated until the cells reached 70% confluency, and then incubated with serum-free medium for 24 hours, which was replaced with EdU-containing 10% FBS and 10% ucPRP and incubated for another 24 hours. Then, the EdU kit instructions (C10310, RiboBio, China) were followed to conduct the experiment. The immunofluorescent signal was assessed by using an Invitrogen™ EVOS™ FL Auto (Nikon Corporation, Japan), and the positivity rate was analyzed by using ImageJ (National Institutes of Health, USA).

### 2.5. Flow Cytometry Analysis

For cell apoptosis analysis, 1 × 10^5^ HucMSCs were inoculated in a 6-well plate. When the cells reached 70% confluency, the serum-free medium was replaced, and the cells were incubated for 24 hours, followed by incubation for 24 hours in medium containing 10% FBS or 10% ucPRP. According to the instructions of the Annexin V-FITC/PI Apoptosis Kit (KGA107, Kegen Biotech, China), 1 × 10^5^ cells were collected, 5 *μ*L Annexin V-FITC and 5 *μ*L PI were added, and the cells were incubated in a dark environment for 15 minutes, followed by immediate assessment by flow cytometry (BD Biosciences, USA); each group was repeated 4 times independently.

### 2.6. Gene Analysis

To observe the effect of ucPRP on the stemness of HucMSCs, 1 × 10^5^ HucMSCs of the P5 generation from different donors were inoculated in a 6-well plate, separately. After the serum-free medium was replaced for 24 hours in the logarithmic growth phase, the medium was refreshed with 10% FBS and 10% ucPRP and cultured for another 24 hours. The collected cells were washed 3 times in cold PBS. RNA was purified according to the instructions of the HiPure Total RNA Mini Kit (R4111-03, Magen, China), and the next experiment was performed. The cDNA obtained by reverse transcription was subsequently used in a 20 *μ*L RT-PCR system. PCR was performed according to the following parameters: a 94°C denaturation for 2 minutes, followed by 40 cycles of 56°C for 30 seconds, 72°C for 35 seconds, and 76°C for 2 seconds. GAPDH was used as the internal control, and all samples were repeated 3 times. See Table 1 for the gene sequence.

### 2.7. Stem Cell Transplantation

The experimental animals were approved by the Animal Care Welfare and Ethics Committee of Guangxi Medical University. Female SD rats (6-8 weeks) were purchased from the Animal Experimental Center of Guangxi Medical University and housed in a warm environment of 24°C. Adequate water and food are given, and the animals experienced 12 h/12 h alternating light and dark every day. Twenty adult female rats were confirmed to have a normal estrus cycle and were randomly assigned to 4 different groups (*n* = 5): the control group, sham group, HucMSC group, and HucMSC+ucPRP group. The POF model was established as described in a previous article. In brief, pulsed administration was used, with the first dose of 50 mg/kg CTX, followed by 8 mg/kg CTX for 15 days of continuous treatment [24]. The HucMSCs were first incubated in serum-free medium for 24 hours and resuspended in PBS twice before transplantation. After chemotherapy treatment, 1 rat in each group was sacrificed, the serum and ovaries were collected, and the remaining rats underwent the previously described orthotopic ovarian transplantation treatment plan. The control group did not receive transplantation. Both ovaries of the HucMSC group received 35 *μ*L physiological serum treatment with 2 × 10^6^ cells, the HucMSC+ucPRP group received treatment with 35 *μ*L ucPRP and 2 × 10^6^ HucMSCs, and the sham group received the same operation but only received treatment with 35 *μ*L physiological serum. After transplantation, 2 rats in each group on D15 and D30 were sacrificed. The serum of each rat was collected and stored at -80°C for subsequent experiments. The ovary was divided into two parts. The left tissue was used to track the HucMSCs. The right panel was used for paraffin embedding for HE, IHC, and TUNEL assessments [25].

### 2.8. HucMSC Tracking

To track the distribution of stem cells in vivo, the PKH26 Red Fluorescent Cell Linker Mini Kit (Sigma-Aldrich, USA) was used to produce PKH26-labeled stem cells, which were used for transplantation. Ovary, heart, liver, spleen, lung, and kidney tissues of D0, D15, and D30 were collected, fixed with an OCT embedding agent, cut into fresh slices (6 *μ*m), fixed in acetone for 15 minutes, stained with DAPI (28718, Solarbio, China) for 15 minutes, and washed twice in PBS. The whole process was performed in a 4°C dark environment, and each slice was immediately recorded with a fluorescence microscope (Nikon Corporation, Japan) in each slice with 4 different 200x fields of view. The positivity rate was qualitatively determined by using ImageJ (National Institutes of Health, USA) [26].

### 2.9. Estrus Cycle and Weight Detection

The weight of the rats was determined at 8 o'clock every day, and a vaginal smear was performed to detect the estrus cycle of the rat. Briefly, a cotton swab containing physiological saline was gently rotated in the rat vagina, and then the collected contents were smeared onto a glass slide. After the slide was dry, Swiss Dye (G1020, Solarbio, China) was used to stain the slide for 1 min. PBS was used to remove excess dye from the slide, and the slides were observed under a microscope (Olympus BX53F, Japan). The estrous cycle of the rats (proestrous, estrous, metestrous, and diestrous) was determined according to previously reported methods [27].

### 2.10. Hormone Determination

The serum of each transplantation group at D-15, D0, D15, and D30 was collected, and the serum hormone content was determined. Briefly, the collected blood was centrifuged at 3000 × *g* for 10 minutes to separate the serum, which was immediately stored at -80°C. The hormone contents of E2, FSH, and AMH (CEA461Ge, CEA830Ra, and CEA228Ra, Cloud-Clone Cope, China) were detected by ELISA.

### 2.11. Ovarian Morphology Assessment and Follicle Count

The collected ovarian tissue was fixed with 4% polymethanol overnight, and the specimens were made into 4 *μ*m sections by paraffin embedding. The four sequence slices of each tissue were collected and used for HE staining. The stained HE sections used the NDP.VIEW image acquisition system. The 2.6.17 software (Hamamatsu Photonics, Japan) was used for observation. The primordial follicles, primary follicles, secondary follicles, and antral follicles were counted using a previously described method [28].

### 2.12. Ovarian Immunohistochemical Staining

Immunohistochemical staining of CD34 (ab81289, Abcam, UK), FSHR (orb213952, Biorbyt, UK), AMH (orb523061, Biorbyt, UK), PCNA (ab29, Abcam, UK), and caspase-3 (ab184787, Abcam, UK) was performed in D15 ovarian tissue. After fixation, the ovarian tissue was embedded into 4 nm thick sections, followed by dewaxing, rehydration, antigen retrieval, blocking, addition of the primary antibodies against CD34, FSHR, AMH, PCNA, and caspase-3, and counterstaining with the secondary antibody and hematoxylin. Finally, the images were collected under a microscopy system (Hamamatsu Photonics, Japan). CD34 staining was located in the ovarian stroma and corpus luteum. FSHR, AMH, PCNA, and caspase-3 were mainly located in the ovarian granulosa cells. Four fields of view were randomly selected for observation at 400x. CD34 was scored using MVD [29]. FSHR, AMH, PCNA, and caspase-3 were scored using the *H* score. According to the *H* score equation, *H*‐score∑Pi (*i* + 1), where *i* is the intensity staining (0 = negative, 1 = weak, 2 = medium, and 3 = strong). Pi is at each intensity (0–100%) percentage of stained cells. The *H* scores were measured in granulosa cells. The sections were independently scored by two pathologists. If there is a difference in the scores, reevaluate the slides and the two observers reached a consensus [30].

### 2.13. TUNEL Staining

To detect the apoptosis of granular cells, an apoptosis kit (11684795910, In Situ Cell Death Detection Kit, Roche, USA) was used to assess D15 granule cell apoptosis. The 4 mm section was deparaffinized and hydrated, incubated in the TUNEL reaction solution at 37°C for 60 minutes, and labeled with DAPI (28718, Solarbio, China) for 10 minutes. Under the microscope field of a 200x fluorescence microscope (Nikon Corporation, Japan), 4 fields were randomly selected to determine the apoptosis rate of the granular cells [31].

### 2.14. Statistical Analysis

All data were analyzed by using SPSS 22.0, and the quantitative data are expressed as the mean ± standard deviation. Data were compared and analyzed by one-way ANOVA and Student's *t*-tests among the different groups, and a statistical graph was generated using Prism 8. Student's *t*-tests are represented by ^+^*P* < 0.05, ^++^*P* < 0.01, ^+++^*P* < 0.001, and ^∆^*P* > 0.05. One-way ANOVA results are represented by ^∗^*P* < 0.05, ^∗∗^*P* < 0.01, ^∗∗∗^*P* < 0.001, and ^#^*P* > 0.05. When *P* was less than 0.05, it was considered statistically significant.

## 3. Results

### 3.1. Identification and Multipotent of Stem Cells

**Figure 1:**
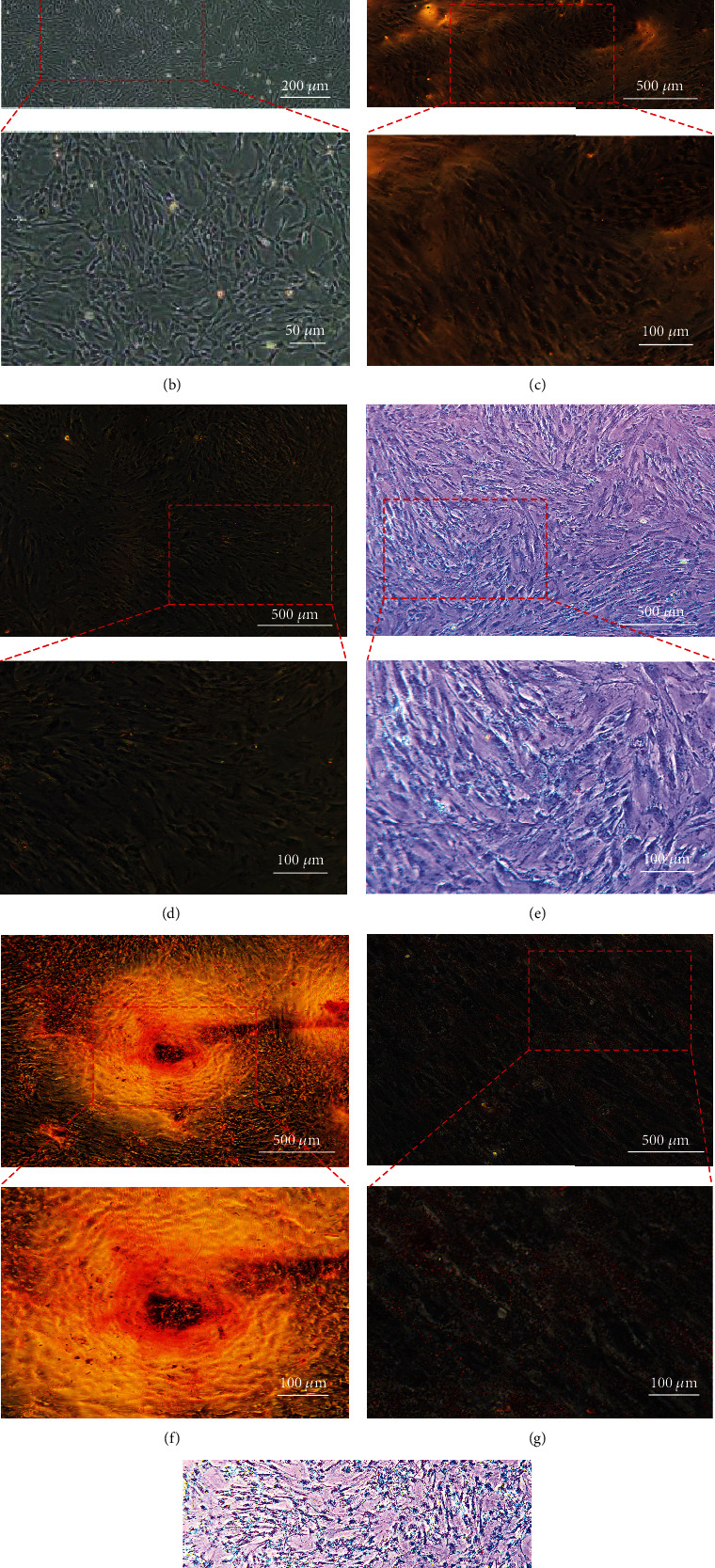
Identification and differentiation potential of stem cells. The surface markers and trilineage differentiation potential of the stem cells were tested. (a) The HucMSCs were positive for CD44, CD73, CD90, and CD105 and negative for CD34, CD45, CD11b, CD14, CD19, CD79*α*, and HLA-DR. (b) Morphology of the stem cells under a light microscope. Scale bar: 200 *μ*m, 50 *μ*m. (c) Alizarin Red staining of undifferentiated stem cells. Scale bar: 500 *μ*m, 100 *μ*m. (d) Oil Red O staining of undifferentiated stem cells. Scale bar: 500 *μ*m, 100 *μ*m. (e) Alcian blue staining of undifferentiated stem cells. Scale bar: 500 *μ*m, 100 *μ*m. (f) After 21 days of incubation in osteogenic differentiation medium, red calcium nodules appeared on Alizarin Red staining. Scale bar: 500 *μ*m, 100 *μ*m. (g) After 21 days of incubation in adipogenic differentiation medium, red fat droplets appeared in stem cells stained with Oil Red O. Scale bar: 500 *μ*m, 100 *μ*m. (h) After 21 days of incubation in the chondrogenic differentiation medium, the acid mucopolysaccharide was stained blue with Alcian blue staining. Scale bar: 500 *μ*m, 100 *μ*m.

We isolated HucMSCs by the tissue block adherence method. On the 3rd day of culture, the HucMSCs showed thin fusiform and fibrous growth under the microscope (Figure 1(b)). The surface markers of the HucMSCs were detected by flow cytometry. The expression levels of CD44, CD73, CD90, and CD105 were positive, and the expression levels of CD34, CD45, CD11b, CD14, CD19, CD79*α*, and HLA-DR were negative (Figure 1(a)). Undifferentiated HucMSCs did not show specific staining (Figures 1(c)–1(e)). Osteogenesis-induced HucMSCs observed red calcium nodules stained with Alizarin Red (Figure 1(f)). Red fat droplets stained with Oil Red O observed in HucMSCs were induced by adipogenesis (Figure 1(g)). Alcian blue-stained blue acid mucopolysaccharide observed in HucMSCs was induced by chondrogenesis (Figure 1(h)). The above results show that we successfully isolated HucMSCs and that they had multipotent.

### 3.2. ucPRP Promotes the Proliferation of the HucMSCs and Reduces Apoptosis

**Figure 2:**
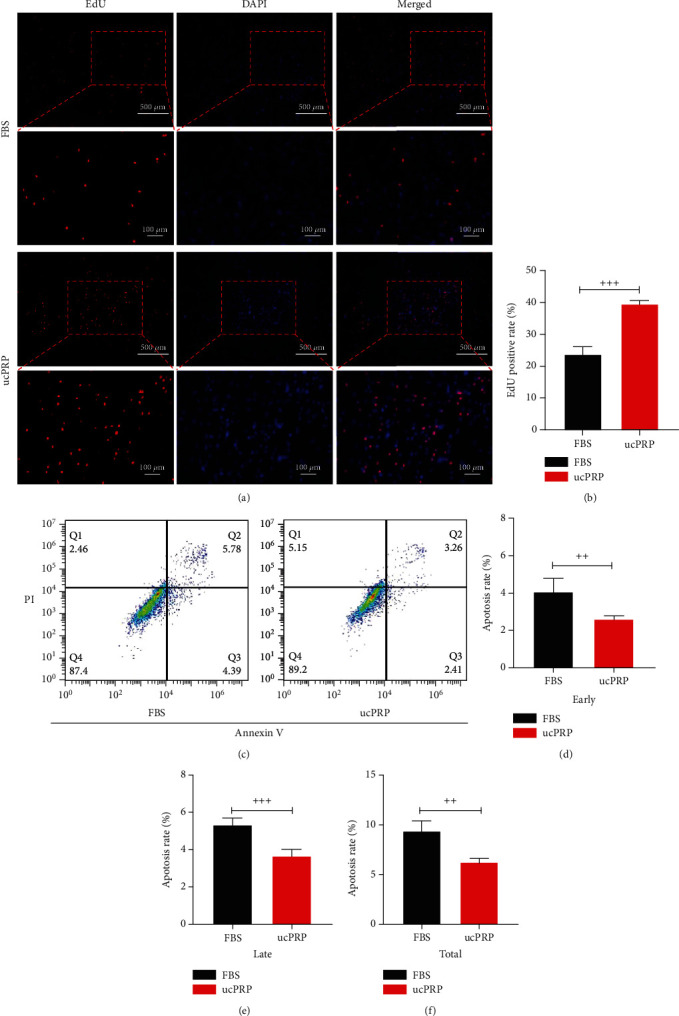
The effect of ucPRP on the proliferation and apoptosis of the HucMSCs. (a) EdU fluorescence after incubation of ucPRP and FBS for 24 hours. Red represents DNA replication, and blue represents the cell nucleus. Scale bar: 500 *μ*m, 100 *μ*m. (b) Numbers of replicated cells were quantified for the indicated groups. (c) Flow cytometry to detect the apoptosis of HucMSCs after incubation with ucPRP and FBS. (d) After 24 hours of stimulation, flow cytometry showed that ucPRP reduced the early apoptosis of HucMSCs. (e) After 24 hours of stimulation, flow cytometry showed that ucPRP reduced the late apoptosis of HucMSCs. (f) After 24 hours of stimulation, flow cytometry showed that ucPRP reduced the total apoptosis of HucMSCs. Data represent the mean ± SEM. ^++^*P* < 0.01, ^+++^*P* < 0.001.

To verify the proliferative effect of ucPRP on HucMSCs, an EdU assay was used. As shown in Figures 2(a) and 2(b), the positive signal in the ucPRP group was significantly higher than that in the FBS group (39.60 ± 1.05 vs. 23.80 ± 2.31, *P* < 0.001). The results showed that ucPRP accelerated the DNA replication process of the HucMSCs and promoted the proliferation of the HucMSCs within 24 h. Subsequently, the Annexin V-FITC/PI Apoptosis Kit was used to further examine the protective effect of ucPRP on HucMSC apoptosis by flow cytometry. As shown in Figures 2(c)–2(f), compared with the physiological serum treatment for 24 hours, the early apoptosis rate of the physiological serum group was 1.57 times that of the ucPRP group (4.07 ± 0.72 vs. 2.60 ± 0.18, *P* = 0.0073), the late apoptosis rate was 1.45 times that of the ucPRP group (5.31 ± 0.39 vs. 3.66 ± 0.35, *P* < 0.001), and the total apoptosis rate was 1.5 times that of the ucPRP group (9.39 ± 1.01 vs. 6.26 ± 0.40, *P* = 0.0012). The results show that the addition of ucPRP reduces the apoptosis level of the HucMSCs.

### 3.3. UcPRP Upregulates the Stemness Genes of the HucMSCs

**Figure 3:**
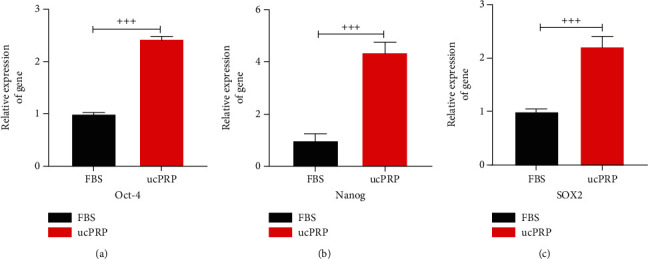
The stemness genes of the P5 generation HucMSCs of different donors were upregulated after ucPRP treatment. (a) Oct-4 gene expression in the HucMSCs after 24 h of ucPRP treatment. (b) Change in Nanog expression after 24 h of ucPRP treatment. (c) Change in SOX2 gene expression after 24 h of ucPRP treatment. Data represent the mean ± SEM. ^+++^*P* < 0.001.

The stemness gene changes in the HucMSCs from the addition of ucPRP were examined by RT-PCR. As shown in Figures 3(a)–3(c), our results showed that after incubation for 24 hours with ucPRP and physiological serum, in the ucPRP group, the stemness genes Oct-4 (2.43 ± 0.05 vs. 1.00 ± 0.02, *P* < 0.001), Sox2 (2.21 ± 0.20 vs. 1.00 ± 0.05, *P* < 0.001), and Nanog (4.37 ± 0.40 vs. 1.00 ± 0.25, *P* < 0.001) in the HucMSCs increased significantly compared with the physiological serum group. This shows that the addition of ucPRP not only does not damage the stemness of HucMSCs but also helps upregulate the stemness genes of the HucMSCs.

### 3.4. HucMSCs and ucPRP Accelerate the Recovery of the Estrus Cycle

**Figure 4:**
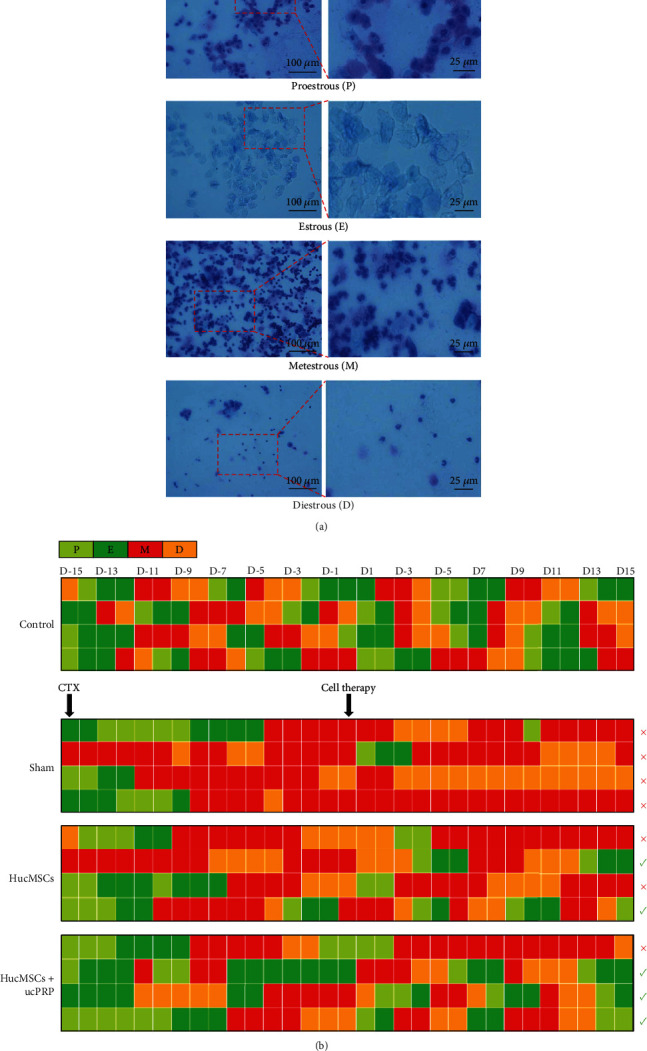
Changes in the estrous cycle of rats over 31 days. (a) Different estrus cycles (proestrous/estrous/metestrous/diestrous) as observed under a light microscope vaginal smear examination. Scale bar: 100 *μ*m, 25 *μ*m. (b) The estrus cycle record. The POF rats in the HucMSC group and the HucMSC+ucPRP group had regular estrus cycles, but the rats in the sham group still retained a chaotic estrus cycle. The red × represents the ongoing chaos in the estrus cycle, and the green √ represents the individuals whose estrus cycle had recovered.

We identified the estrous cycle of experimental rats (*n* = 4) as the four stages of proestrous, estrous, metestrous, and diestrous by the vaginal smear method (Figure 4(a)) and evaluated the estrous cycle changes over 31 days (Figure 4(b)). The estrus cycle of normal rats is 3 to 5 days, and POF rats with a disordered cycle mean remain in the metestrous and diestrous leukocitary phases. Once the rat reached regular proestrous and estrous, it means that the POF rat restores regular cycling [27]. In our research, the rats in the control group completed an average of 5.3 ± 0.3 cycles. After modeling, all the cycles of the CTX-treated rats were destroyed, and there was no improvement in the cycles of the rats treated with physiological serum. However, 50% of the rats recovered a regular estrus cycle after HucMSC treatment with an average of 1.5 ± 1.1 cycles, and in the HucMSCs combined with the ucPRP treatment group, 75% of the rats showed an average of 2.0 ± 0.8 cycles. The recovery of the estrous cycle of rats treated with HucMSCs+ucPRP occurred earlier than that of the rats treated with the HucMSCs alone.

### 3.5. Distribution and Fate of HucMSCs In Vivo

**Figure 5:**
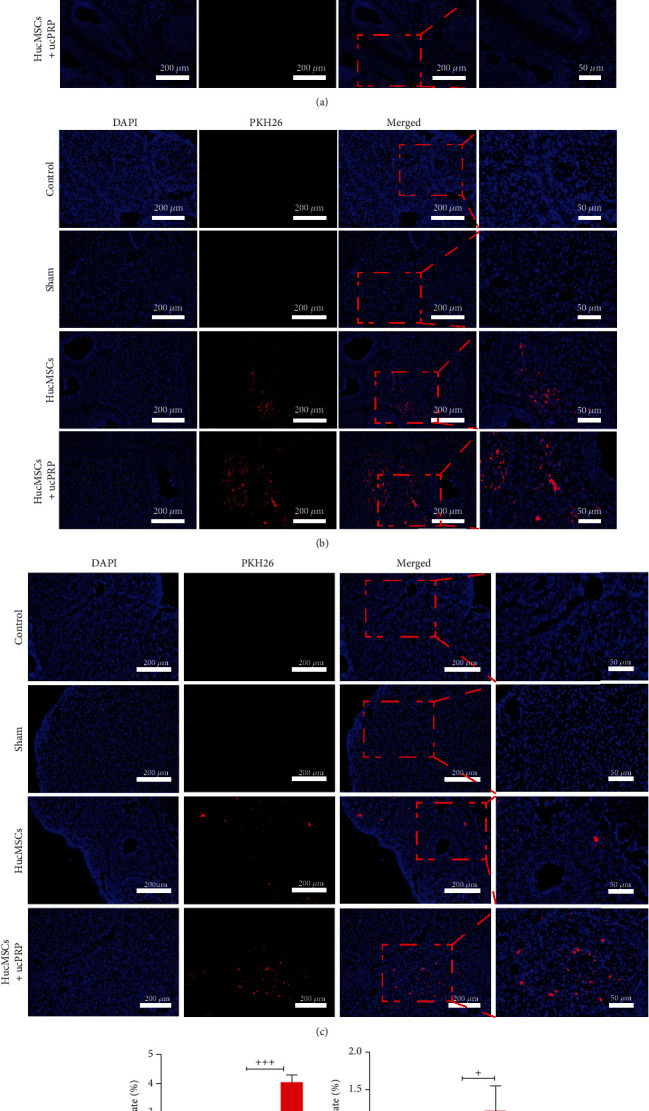
ucPRP enables more HucMSCs to survive in the ovaries. Red represents HucMSCs labeled with PKH26, and blue represents cell nuclei. (a) The rat ovary did not express any red fluorescence on D0. Scale bar: 200 *μ*m, 50 *μ*m. (b) Fifteen days after transplantation, the HucMSCs were mainly distributed in the ovarian stroma and the follicular membrane. Scale bar: 200 *μ*m, 50 *μ*m. (c) The retention of the HucMSCs in the ovary 30 days after transplantation. Scale bar: 200 *μ*m, 50 *μ*m. (d) Statistical analysis of the fluorescence positivity rate of the HucMSCs on D15. (e) Statistical analysis of the fluorescence positivity rate of the HucMSCs at D30. Data represent the mean ± SEM. ^+^*P* < 0.05, ^+++^*P* < 0.001.

We used PKH26 to label HucMSCs and tracked the distribution and fate of the HucMSCs at D0 (Figure 5(a)), D15 (Figure 5(b)), and D30 (Figure 5(c)) after transplantation. In the heart, liver, spleen, lung, and kidney, no migration of HucMSCs was observed (Figure S1). The HucMSCs survived in the ovaries for at least 30 days. The HucMSCs were mainly located around the ovarian stroma and the follicular membrane. As shown in Figures 5(d) and 5(e), no fluorescence was observed at D0. The fluorescence area of the D15 HucMSC+ucPRP group after transplantation was significantly higher than that of the HucMSCs group alone (4.06 ± 0.25 vs. 0.98 ± 0.06, *P* < 0.001). The difference at D30 was reduced by 1.7-fold (1.23 ± 0.32 vs. 0.71 ± 0.24, *P* = 0.041). Our results suggest that the addition of ucPRP allows a greater number of HucMSCs to be retained in the body.

### 3.6. Weight and Hormone Changes

**Figure 6:**
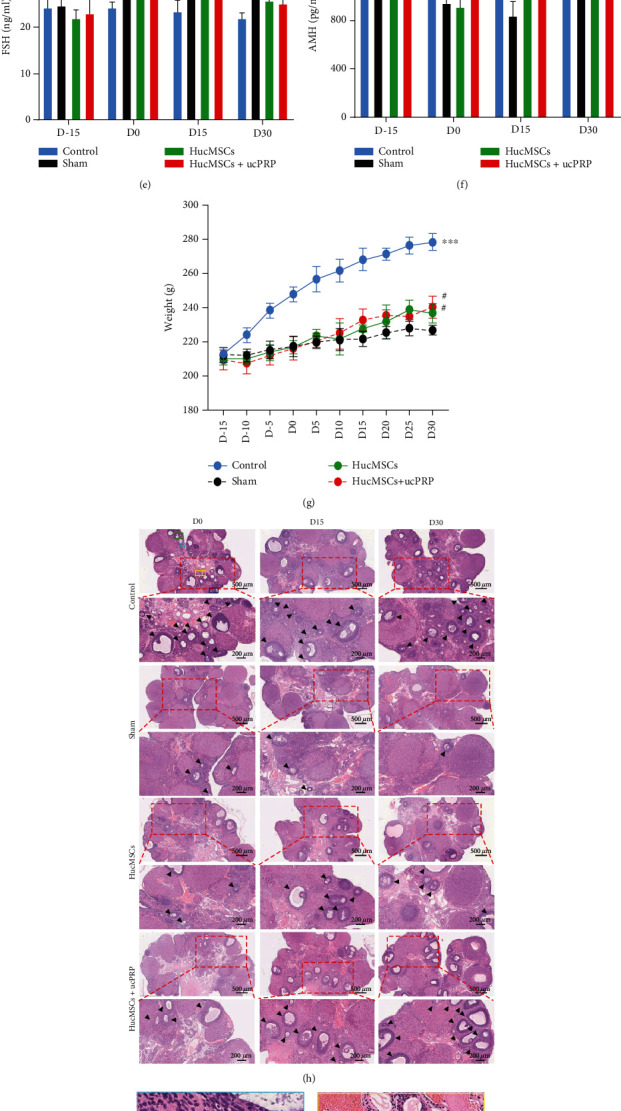
HucMSCs+ucPRP promote the recovery of ovarian endocrine function and follicular development. (a) Rat ovary and uterus after CTX treatment. (b) Rat ovarian tissue shrinkage after POF treatment. (c) Stem cells transplanted into the ovary. (d) Changes in serum E2. (e) Changes in serum FSH. (f) Changes in serum AMH. (g) Changes in the body weight of the rats. (h) HE staining of pathological sections of ovarian tissue. The black arrow points to the counted follicle. Scale bar: 500 *μ*m, 200 *μ*m. The pictures of healthy rat ovaries in the boxes of different colors marked in the D0 control group are enlarged and marked as (i) primordial follicle, (j) primary follicle, (k) primary follicle, and (l) antral follicle, respectively. Oo: oocyte, Gc: granulosa cell. Scale bar: 50 *μ*m. (m) Statistical analysis of follicle counts at all levels of the D15 ovary. Data represent the mean ± SEM. ^#^*P* > 0.05, ^∗^*P* < 0.05, ^∗∗^*P* < 0.01, and ^∗∗∗^*P* < 0.001.

We confirmed that after 16 days of CTX treatment, the ovaries of the POF rats decreased in size (Figures 6(a) and 6(b)), and we monitored the changes in body weight and hormones of POF rats after transplantation of HucMSCs and ucPRP (Figures 6(c)–6(g)). During the 30-day follow-up period, the body weight of all rats increased, but the CTX treatment resulted in slower weight gain in the rats. Compared with the sham group, the HucMSCs and the HucMSC+ucPRP transplantation groups promoted weight gain in the rats, but there was no statistical significance (*P* > 0.05). The concentration of sex hormones was monitored for up to 45 days. In all treatment groups that had undergone CTX treatment, E2, AMH, and FSH were negatively affected. On D15 (34.86 ± 7.22 vs. 23.23 ± 5.05, *P* = 0.040) and D30 (39.05 ± 2.45 vs. 21.30 ± 3.24, *P* = 0.044), E2 in the HucMSC+ucPRP group increased significantly with the increase in transplantation time, and the HucMSC group showed the same trend (30.35 ± 5.33 vs. 23.23 ± 5.05 (*P* > 0.05) and 32.23 ± 5.50 vs. 21.30 ± 3.24 (*P* > 0.05), respectively), but there was no difference compared with the sham group. On D15 and D30, the AMH and FSH of the HucMSC group and the HucMSC+ucPRP group showed the same recovery trend. In particular in the D15 sham operation group, there were significant differences compared with the HucMSC group (830.99 ± 117.2 vs. 1048.26 ± 90.09 (*P* = 0.043) and 32.19 ± 3.12 vs. 25.86 ± 1.71 (*P* = 0.041), respectively) and the HucMSC+ucPRP group (830.99 ± 117.2 vs. 1092.07 ± 57.05 (*P* = 0.036) and 32.19 ± 3.12 vs. 26.46 ± 1.31 (*P* = 0.024), respectively). The transplantation of the HucMSCs alone restored the endocrine function of the ovary, and the combined application of HucMSCs and ucPRP enhanced this effect.

### 3.7. Combined Transplantation of HucMSCs and ucPRP Improves the Development of Follicles

We collected ovarian tissues at D0, D15, and D30 to observe follicular development (Figures 6(h)–6(l)), and the HE tissue sections revealed that the number of follicles at all levels in the group undergoing 16 days of CTX treatment decreased. On D15 after transplantation, the primordial follicles (12.5 ± 0.7 vs. 6.0 ± 1.4, *P* = 0.012) and primary follicles (7.5 ± 0.7 vs. 3.5 ± 0.7, *P* = 0.043) in the HucMSC group started to develop compared with those in the sham group. More primordial follicles and primary follicles developed in the HucMSC+ucPRP group (18.5 ± 3.5 vs. 6.0 ± 1.4 (*P* = 0.003) and 8.5 ± 0.7 vs. 3.5 ± 0.7 (*P* = 0.020), respectively) in comparison to the HucMSC group. In particular, the number of primordial follicles in the HucMSC+uPRP group was 1.48 times that in the HucMSC group. Transplantation of HucMSCs and HucMSCs+ucPRP also increased the number of secondary follicles and antral follicles, but there was no difference from the sham group. The morphology of the HucMSC group and HucMSC+ucPRP group on D30 showed the same trend as that on D15, while the follicles in the sham group were still poorly developed. Our results show that HucMSC transplantation can improve the development of follicles and that the combined transplantation of HucMSCs and ucPRP has better benefits.

### 3.8. Combined Transplantation of HucMSCs and ucPRP Promotes Angiogenesis and Expression of Follicular Development Markers

**Figure 7:**
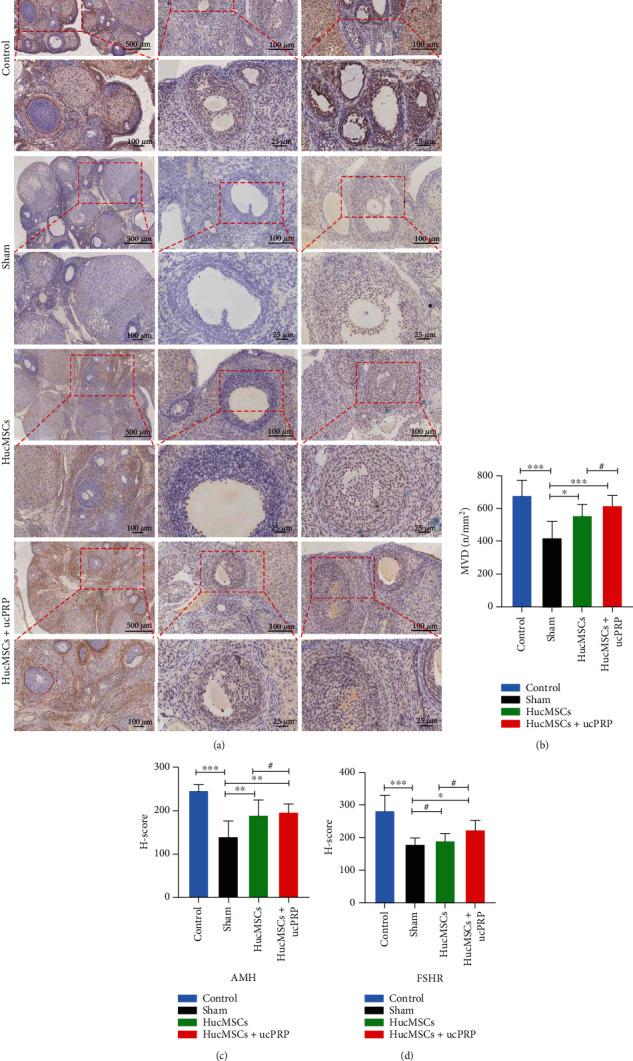
The combined transplantation of HucMSCs and ucPRP accelerates the recovery of ovarian angiogenesis and follicular development marker expression. (a) The expression of the D15 ovarian angiogenesis marker CD34 and the follicular development markers AMH and FSHR. (b) CD34 staining was located in the ovarian stroma and corpus luteum. The MVD score assessed the changes in the expression of CD34 ovarian angiogenesis markers. Scale bar: 500 *μ*m, 100 *μ*m. (c) AMH mainly located in the ovarian granulosa cells. The *H* score assessed the changes in the expression of the AMH ovarian development marker. Scale bar: 100 *μ*m, 25 *μ*m. (d) FSHR located in the ovarian granulosa cells. The *H* score evaluated the expression changes in the FSHR ovarian development marker. Scale bar: 100 *μ*m, 25 *μ*m. Data represent the mean ± SEM. ^#^*P* > 0.05, ^∗^*P* < 0.05, ^∗∗^*P* < 0.01, and ^∗∗∗^*P* < 0.001.

To verify the expression of the ovarian angiogenesis marker CD34 and the follicular development markers AMH and FSHR (Figure 7(a)), D15 sections after transplantation were stained. CD34 was mainly expressed in the ovarian stroma. Compared with the sham group, the angiogenesis of the HucMSC (555.0 ± 71.5 vs. 422.2 ± 99.9, *P* = 0.019) and HucMSC+ucPRP (615.8 ± 65.5 vs. 422.2 ± 99.9, *P* < 0.001) groups increased. Compared with the HucMSC+ucPRP group and the HucMSC group, the MVD score increased by 1.1 times, but there was still a certain difference compared with the control group (Figure 7(b)). AMH was mainly expressed in the primary follicles and the antral follicles; expression in the HucMSC+ucPRP group was second only to that of the control group and was higher than that of the sham group (196.9 ± 18.89 vs. 140.3 ± 36.39, *P* = 0.002), and the expression in the HucMSC group was also higher than that of the sham group (189.8 ± 35.06 vs. 140.3 ± 36.39, *P* = 0.008) (Figure 7(c)). FSHR was mainly expressed in the antral follicles and the preovulatory follicles. The control, HucMSC, and HucMSC+ucPRP groups were all higher than the sham group, but only the control (283.8 ± 45.7 vs. 180.0 ± 19.27, *P* < 0.001) group and the HucMSC+ucPRP group (225.5 ± 27.8 vs. 180.0 ± 19.27, *P* = 0.032) were significantly different compared with the sham group (Figure 7(d)). For CD34, AMH, and FSHR, the expression intensity in the HucMSC+ucPRP group was slightly higher than that of the HucMSCs group and closer to that of the control group. Our results show that the combined transplantation of HucMSCs and ucPRP has advantages in ovarian angiogenesis and follicular development.

### 3.9. Combined Transplantation of HucMSCs and ucPRP Promotes the Proliferation of Ovarian Granulosa Cells and Inhibits Apoptosis

**Figure 8:**
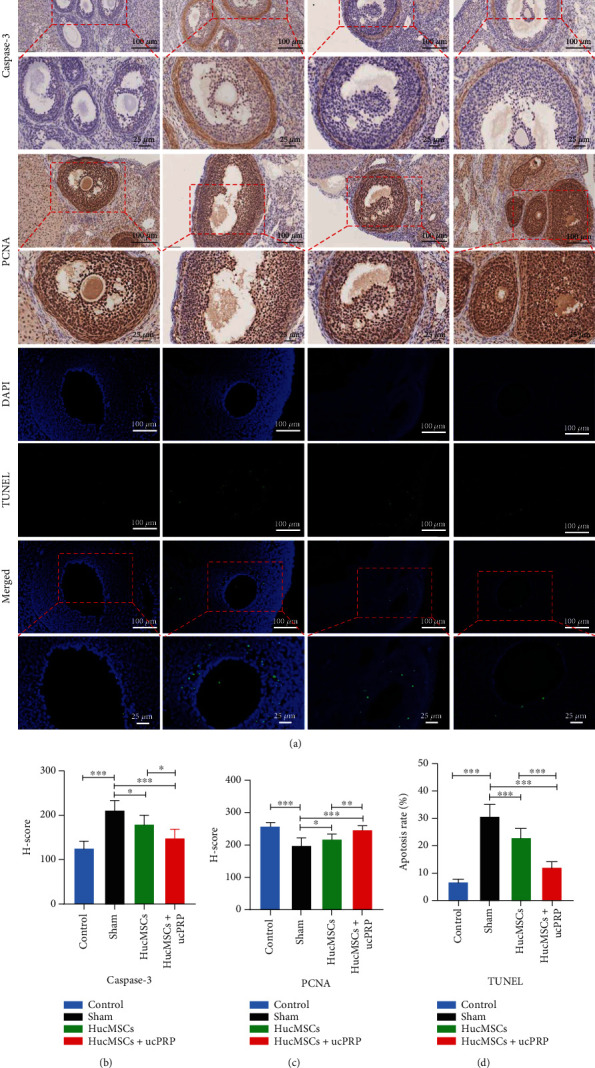
HucMSCs+ucPRP promote the proliferation of ovarian granulosa cells and reduce apoptosis. (a) Immunohistochemical examination of the expression of the granulosa cell proliferation marker PCNA and the apoptosis marker caspase-3 and TUNEL staining examination of granulosa cell apoptosis. (b) Caspase-3 mainly located in the ovarian granulosa cells. *H* score analysis of caspase-3, a marker of granulosa cell apoptosis. Scale bar: 100 *μ*m, 25um. (c) PCNA mainly located in the ovarian granulosa cells. *H* score analysis of the granular cell proliferation marker PCNA. Scale bar: 100 *μ*m, 25 *μ*m. (d) TUNEL staining to detect the apoptosis of ovarian granulosa cells. Scale bar: 100 *μ*m, 25 *μ*m. Green represents apoptotic cells, and blue represents cell nuclei. Data represent the mean ± SEM. ^∗^*P* < 0.05, ^∗∗^*P* < 0.01, and ^∗∗∗^*P* < 0.001.

To observe the effect of HucMSCs+ucPRP on the proliferation and apoptosis of ovarian granulosa cells on D15, we assessed the expression of the proliferation protein PCNA and of the apoptotic protein caspase-3. The apoptosis of granulosa cells was detected by TUNEL staining (Figure 8(a)). In the HucMSC (178.8 ± 21.7 vs. 210.0 ± 23.9, *P* = 0.029) and HucMSC+ucPRP (147.5 ± 21.0 vs. 210.0 ± 23.9, *P* < 0.001) transplantation groups compared with the sham group, the expression of ovarian granulosa cell apoptosis protein caspase-3 was downregulated (Figure 8(b)), and caspase-3 in the HucMSC group was 1.21 times that of the HucMSC+ucPRP group (178.8 ± 21.7 vs. 147.5 ± 21.0, *P* = 0.029). Compared with the sham group, in the HucMSCs (220.6 ± 12.9 vs. 198.8 ± 24.9, *P* = 0.047) and the HucMSC+ucPRP (248.8 ± 11.6 vs. 198.8 ± 24.9, *P* < 0.001) groups, proliferation protein PCNA expression was upregulated, with the HucMSC+ucPRP group showing a better proliferation trend than the HucMSC group (248.8 ± 11.6 vs. 220.6 ± 12.9, *P* = 0.007) (Figure 8(c)). TUNEL staining revealed that after CTX treatment, a large number of granular cells began to undergo apoptosis, but local transplantation of HucMSCs (23.1 ± 3.4 vs. 30.9 ± 4.2, *P* < 0.001) and HucMSC+ucPRP (12.15 ± 2.2 vs. 30.9 ± 4.2, *P* < 0.001) protected the granulosa cells from apoptosis, and that the HucMSC+ucPRP group showed a stronger protective effect than the transplantation of HucMSCs alone (12.15 ± 2.2 vs. 23.1 ± 3.4, *P* < 0.001) (Figure 8(d)). Our results show that HucMSCs and ucPRP synergistically enhance the proliferation and antiapoptotic ability of granulosa cells.

## 4. Discussion

Approximately 1% of 40-year-old women suffer from POF. POF may cause osteoporosis, cardiovascular disease, and even infertility, which seriously affects women's physical and mental health [32, 33]. At present, HRT is mainly used to treat estrogen deficiency, but it cannot trigger ovulation. At the same time, long-term HRT treatment increases the risk of breast cancer and endometrial cancer [34]. The potential of MSCs in the treatment of POF has been reported in recent years [26, 35]. However, the autologous transplantation of bone marrow mesenchymal stem cells (BMSCs) and adipose mesenchymal stem cells (ADSCs) has problems such as additional surgical operations and poor function of aging tissues [36, 37]. Human embryonic stem cells (ESCs) still have ethical constraints [38]. HucMSCs, which lack these limitations, are considered to be the best seed cells for the treatment of POF. However, the effectiveness of HucMSCs alone in the treatment of POF is still controversial. ucPRP is derived from naive umbilical cord tissue, has a wide range of sources and more abundant growth factors than peripheral blood, and is a safe and effective substitute for patients with contraindications to autologous PRP [20, 39]. A recent clinical study showed that ucPRP has a better effect than peripheral blood PRP for the treatment of endometrial injury infertility [40]. However, the beneficial effects of combined transplantation of HucMSCs and ucPRP in POF rats have not been discussed.

For the first time, we observed the biological activity changes of activated ucPRP on HucMSCs in vitro. We further confirmed that ucPRP can promote the proliferation and antiapoptotic ability of HucMSCs [23]. The antiapoptotic ability may be achieved by improving energy metabolism. PRP may upregulate the mitochondrial ATP rate-limiting enzyme 5-ATP synthase in MSCs. The production of ATP helps cells resist metabolic stress in extreme environments [41]. Oct-4, Nanog, and SOX2 are important genes for stem cell multipotent maintenance [42]. In our study, the addition of ucPRP did not harm the expression of multipotent genes in HucMSCs. Overall, ucPRP is a beneficial transplantation additive for HucMSCs.

Transplantation of HucMSCs with a low survival rate in the ovaries may lead to little effect on POF. Improving the transplantation environment in the ovaries plays a decisive role in the fate of transplanted HucMSCs [43]. We observed the fate and distribution of HucMSCs labeled with PKH26. HucMSCs remained in the ovary for at least 30 days. However, no migrating HucMSCs were found in other organs: heart, liver, spleen, lung, and kidney, which may be due to orthotopic ovarian stem cell transplantation. The number of HucMSCs+ucPRP in the ovary was significantly increased compared with that of HucMSCs transplanted alone. These findings emphasize the hypothesis that ucPRP may protect the HucMSCs from apoptosis in the hostile environment of transplantation in vivo, leading to the survival of more HucMSCs. In our study, the HucMSCs were mainly located in the ovarian stroma and follicular membrane, and it is possible that the ovarian granulosa cells were not directly benefited [26]. However, it has also been reported that BMSCs can home to ovarian granulosa cells [35]. This may be due to the difference in the homing ability and differentiation ability of mesenchymal stem cells from different sources [44]. The specific mechanism still needs further study.

We established a POF rat model treated with CTX. After this treatment, weight loss, ovarian shrinkage, negatively altered sex hormones, and a disordered estrus cycle appeared. There was a lack of primordial follicles, primary follicles, secondary follicles, and antral follicles in the ovarian pathology [25]. CTX is fatally toxic to cellular DNA, such that the development of the primordial follicles remains in meiosis stage I and can also induce granular cell apoptosis and stop follicle development [45]. In our study, after the transplantation of HucMSCs+ucPRP and HucMSCs, all levels of follicles began to develop, especially primordial follicles and primary follicles. At 30 days after transplantation, the weight of the HucMSC+ucPRP and HucMSC group increased slightly and still showed a specific difference with the control group, which may be attributed to the systemic injury caused by CTX, and local transplantation alone could not improve the systemic condition. In our results, some POF mice in the HucMSC+ucPRP group and the HucMSC group appeared to have regular cycles, the E2 and AMH showed different degrees of recovery, the FSH continued to decline, and the ovarian endocrine function in the HucMSC+ucPRP group recovered earlier than that of the HucMSC group, similar to what has been observed in previous studies [24, 46]. In short, the combined transplantation of HucMSCs and ucPRP effectively improved the endocrine function of the ovary, and a greater number of follicles that had stopped developing were rescued.

Neovascularization of the ovary provides the necessary guarantees for follicular development, such as nutrition, hormones, and oxygen. In particular, for primordial follicles and primary follicles, because the vascular network has not been established, they can only rely on the blood vessels in the ovarian stroma to supply oxygen and nutrients [47, 48]. HucMSCs+ucPRP synergistically increased the expression of CD34, a specific marker of hematopoietic stem and vascular progenitor cells [49], accelerated the regeneration of ovarian blood vessels, and improved the microenvironment for follicular growth. FSH, as an important hormone that promotes the proliferation of granulosa cells, is dependent on its combination with FSHR. The cessation of follicle development caused by the decreased sensitivity of FSHR is closely related to the occurrence of POF [50]. AMH is an important factor for follicular recruitment, and AMH usually also indicates the reserve of the ovaries [51]. A loss of AMH can lead to overrecruitment of follicles, and AMH can also affect the sensitivity of primordial follicles to FSHR [52]. In our study, HucMSCs+ucPRP achieved better benefits in rebuilding the ovarian vascular network and restoring the expression of AMH and FSHR, the key factors for follicular development, compared to HucMSCs alone.

The growth of granulosa cells determines the fate of follicles [53]. PCNA as a cell cycle-related protein plays a key role in DNA replication and repair [54]. CTX induced a decrease in the expression of PCNA in rat ovarian granulosa cells and increased apoptosis in ovarian granulosa cells. Interestingly, the combined transplantation of HucMSCs and ucPRP upregulated the expression of PCNA, led to granulosa cell proliferation, and induced follicles to begin to develop normally. Caspase-3 is a member of the caspase family. Caspase-3 can activate DNase in the nucleus to cause DNA fragmentation and become the executor in the process of cell apoptosis [55, 56]. Normally developing granulosa cells inhibit the expression of caspase-3 by maintaining the upregulation of X-linked inhibitor of apoptosis protein (XIAP) [57]. In contrast to PCNA, the transplantation of HucMSCs and HucMSCs+ucPRP downregulated the expression of caspase-3 and saved the abnormal atresia of the developing follicles. These results further confirmed that the combined transplantation of HucMSCs and ucPRP accelerated the proliferation of ovarian granulosa cells and reduced the apoptosis of granulosa cells induced by CTX. These mechanisms may be caused by the paracrine pathway, and further studies are needed to confirm this hypothesis. In the future, we will investigate the effect of HucMSCs combined with ucPRP on the CD34 of ovarian endothelial cells and the AMH, FSHR, PCNA, and caspase-3 of granulosa cells by the paracrine pathway at the cellular level.

## 5. Conclusion

In summary, this study found that transplantation of HucMSCs combined with ucPRP improved the ovarian function of POF rats, that ucPRP increased the survival rate of HucMSC transplantation, and that the implanted HucMSCs improved ovarian endocrine function and promoted follicular development by promoting ovarian angiogenesis and reducing granulosa cell apoptosis. For the first time, we confirmed the beneficial effects of combining HucMSCs and ucPRP in the treatment of POF and provided a new cell transplantation treatment plan for clinical POF patients.

## Figures and Tables

**Table 1:** Sequences of PCR primers and length of products.

Gene	Sequence of primer (5′-3′)	Length of product
GADPH	Forward: GTGGACCTGACCTGCCGTCTAG	149
	Reverse: GAGTGGGTGTCGCTGTTGAAGTC	
Oct-4	Forward: GTGGTCCGAGTGTGGTTCTGTAAC	90
	Reverse: CCCAGCAGCCTCAAAATCCTCTC	
Nanog	Forward: GATGCAAGAACTCTCCAACATC	178
	Reverse: CTGGTGGTAGGAAGAGTAAAGG	
SOX2	Forward: CAGCATGTCCTACTCGCAGCAG	117
	Reverse: CTGGAGTGGGAGGAAGAGGTAACC	

## Data Availability

The authors declare that all the data supporting the findings in this study are available from the corresponding author through email on reasonable request.

## Supplementary Materials

Supplementary Materials Figure S1: the distribution of HucMSCs in the rat heart, liver, spleen, lung, and kidney on D0, D15, and D30 after stem cell transplantation.
