# Supplementary material for: Activated Human Umbilical Cord Blood Platelet-Rich Plasma Enhances the Beneficial Effects of Human Umbilical Cord Mesenchymal Stem Cells in Chemotherapy-Induced POF Rats
Source: Stem Cells Int. 2021 Oct 25;2021:8293699. doi: 10.1155/2021/8293699 (PMC8560297; doi:10.1155/2021/8293699)
Supplement: Supplementary Materials — Figure S1: the distribution of HucMSCs in the rat heart, liver, spleen, lung, and kidney on D0, D15, and D30 after stem cell transplantation. [file 8293699.f1.pdf]

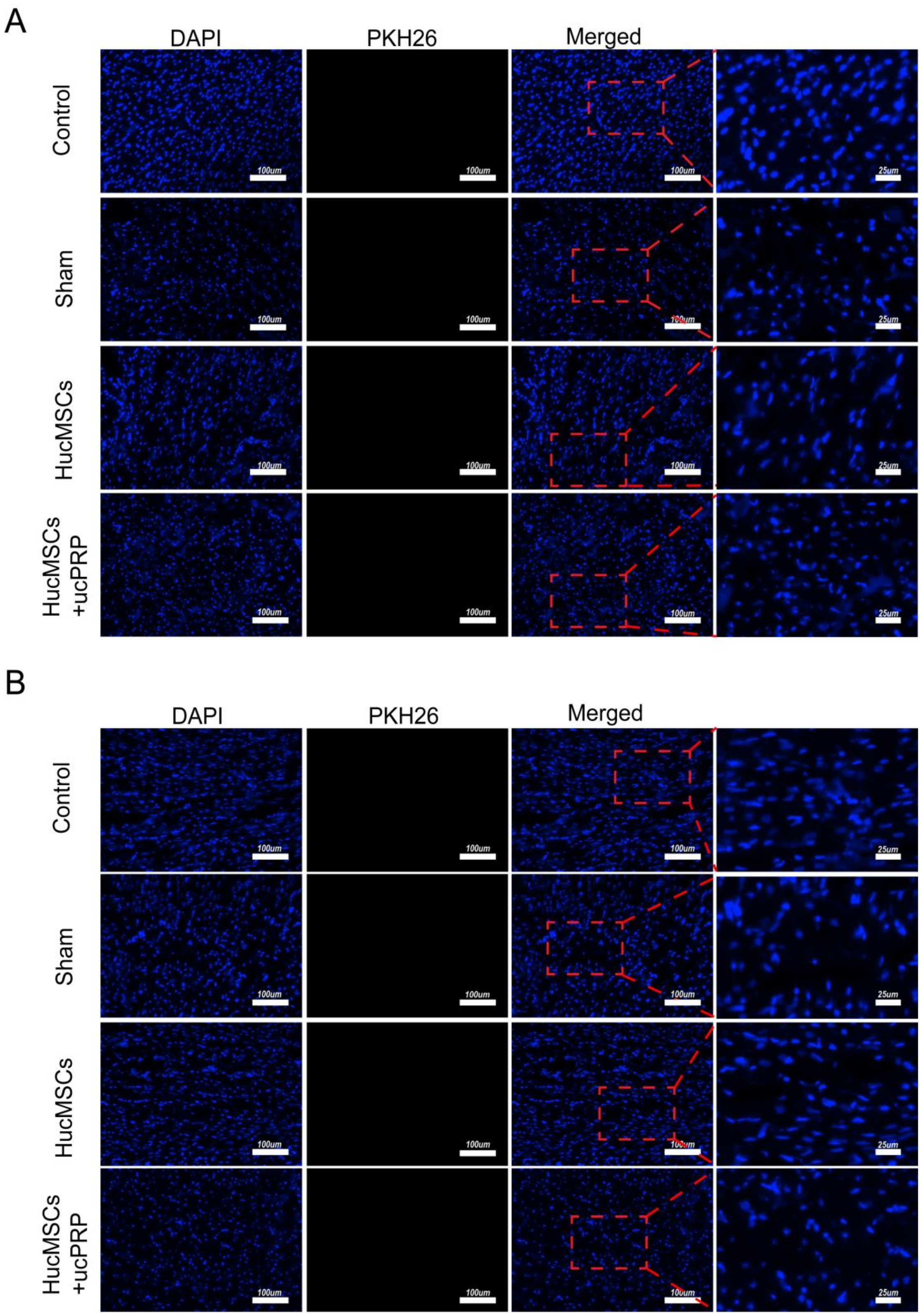

A
DAPI
PKH26
Merged
Control
Sham
HucMSCs
HucMSCs +ucPRP
100um
25um
B
DAPI
PKH26
Merged
Control
Sham
HucMSCs
HucMSCs +ucPRP
100um
25um

1

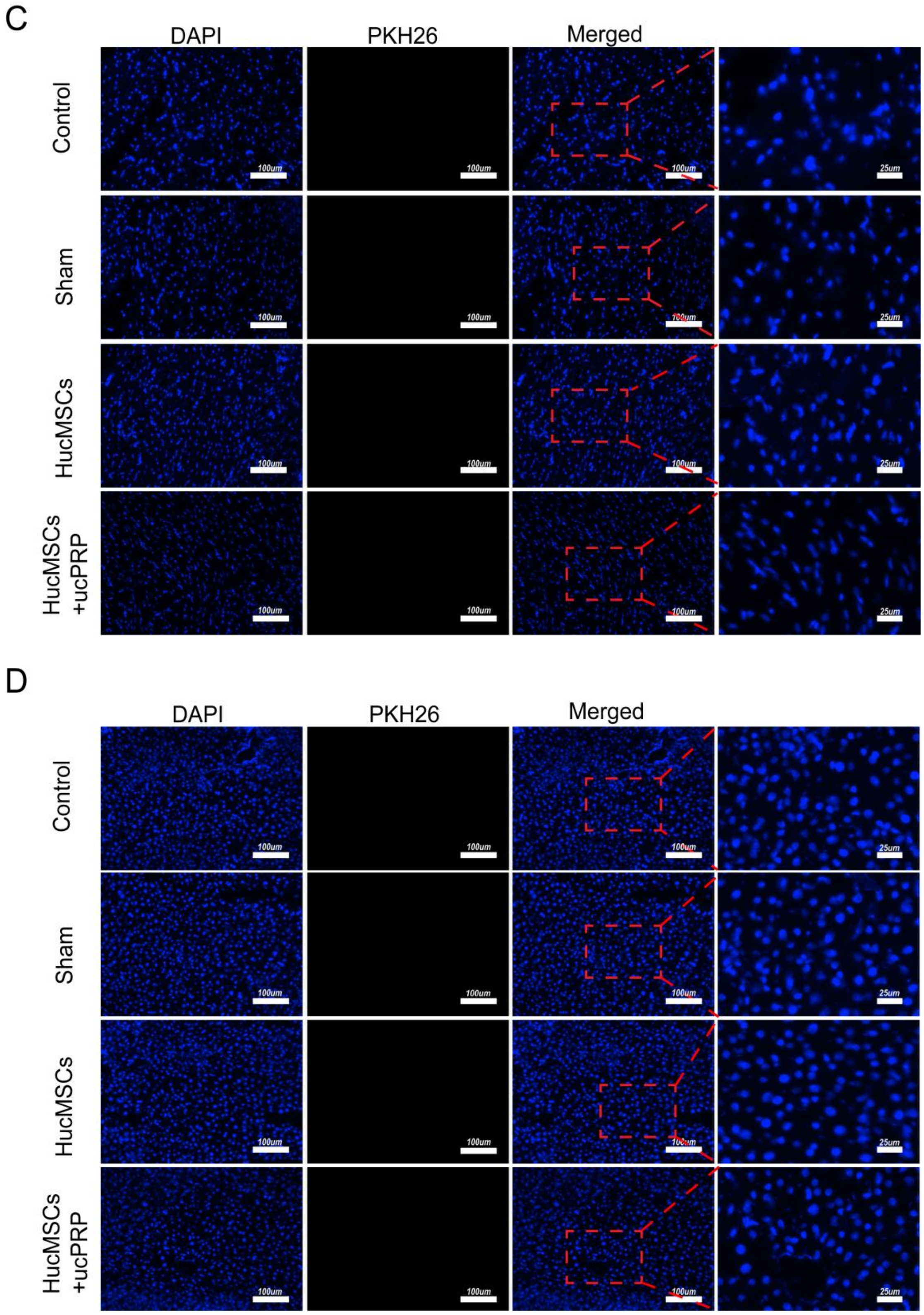
C
DAPI
PKH26
Merged
Control
Sham
HucMSCs
HucMSCs +ucPRP
100um
25um
D
DAPI
PKH26
Merged
Control
Sham
HucMSCs
HucMSCs +ucPRP
100um
25um

2

E

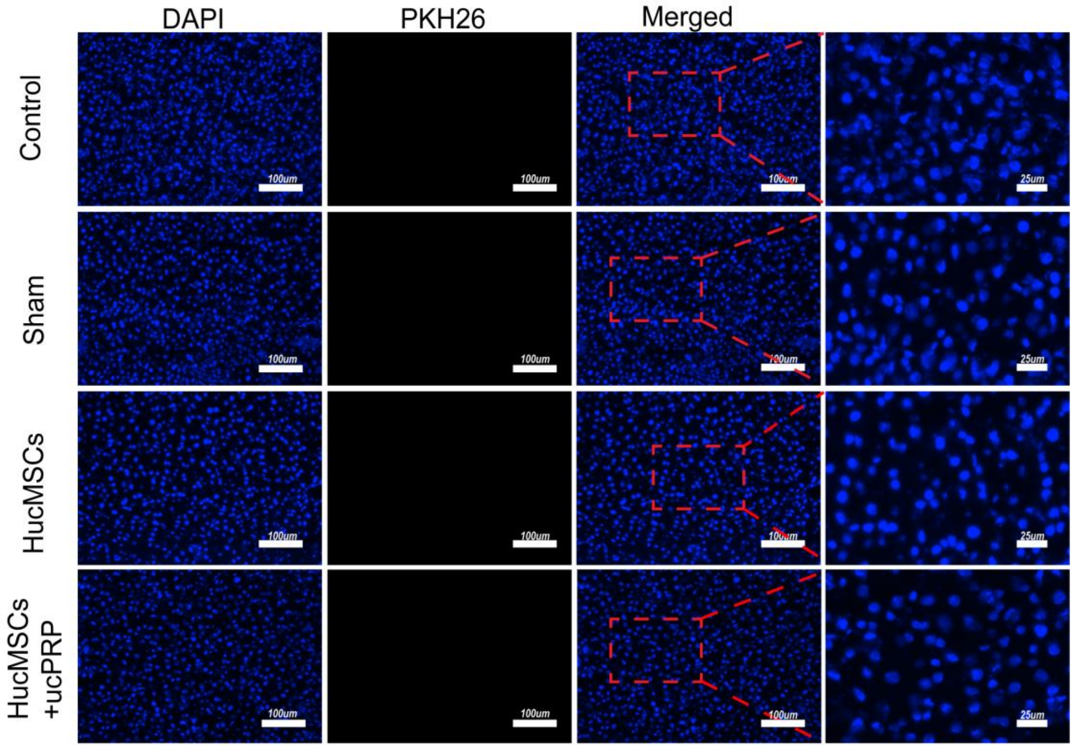
DAPI
PKH26
Merged
Control
Sham
HucMSCs
HucMSCs +ucPRP
100um
25um

F

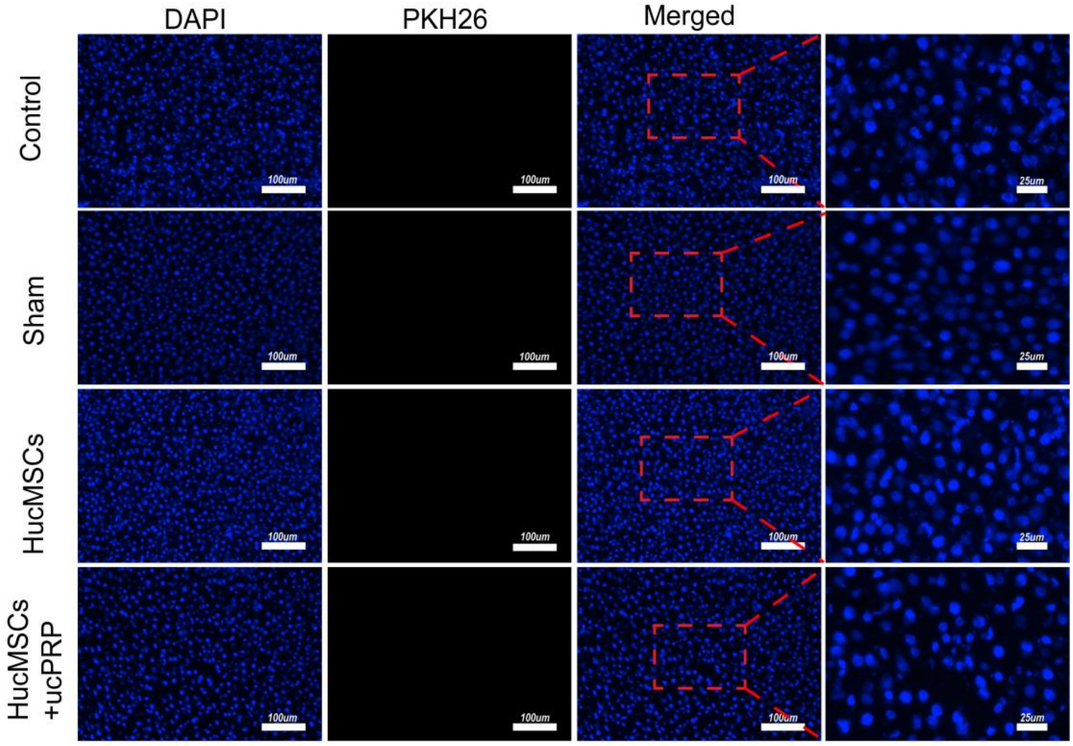
DAPI
PKH26
Merged
Control
Sham
HucMSCs
HucMSCs +ucPRP
100um
25um

G

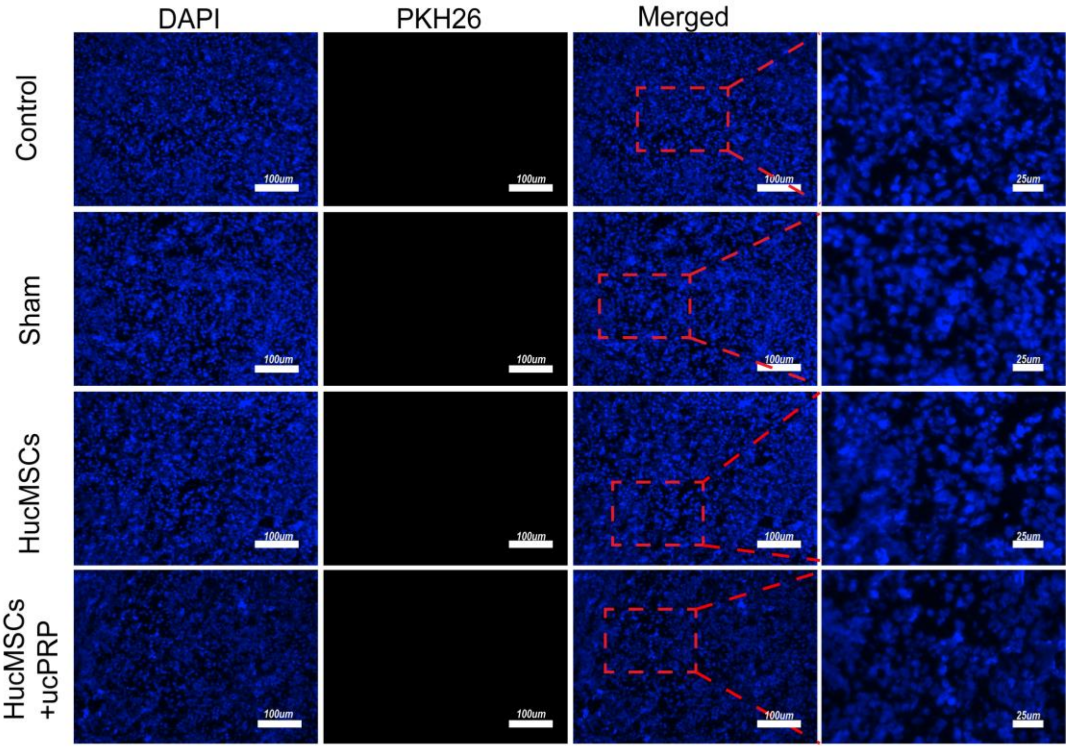
DAPI
PKH26
Merged
Control
Sham
HucMSCs
HucMSCs +ucPRP
100um
25um

H

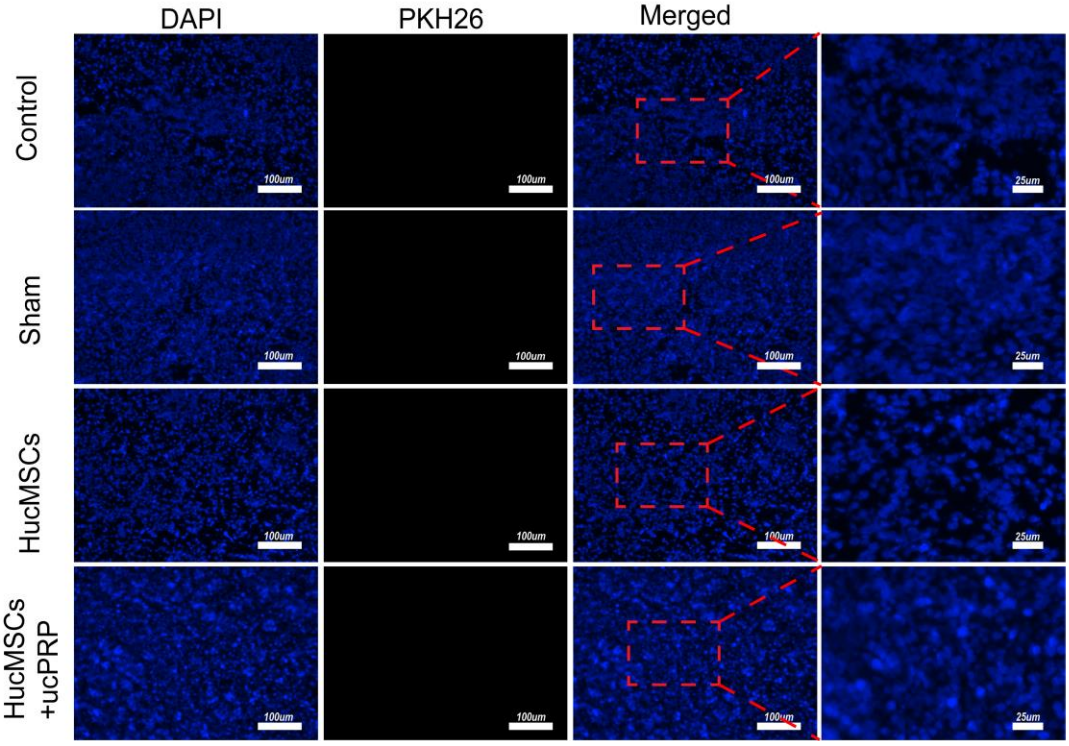
DAPI
PKH26
Merged
Control
Sham
HucMSCs
HucMSCs +ucPRP
100um
25um

I

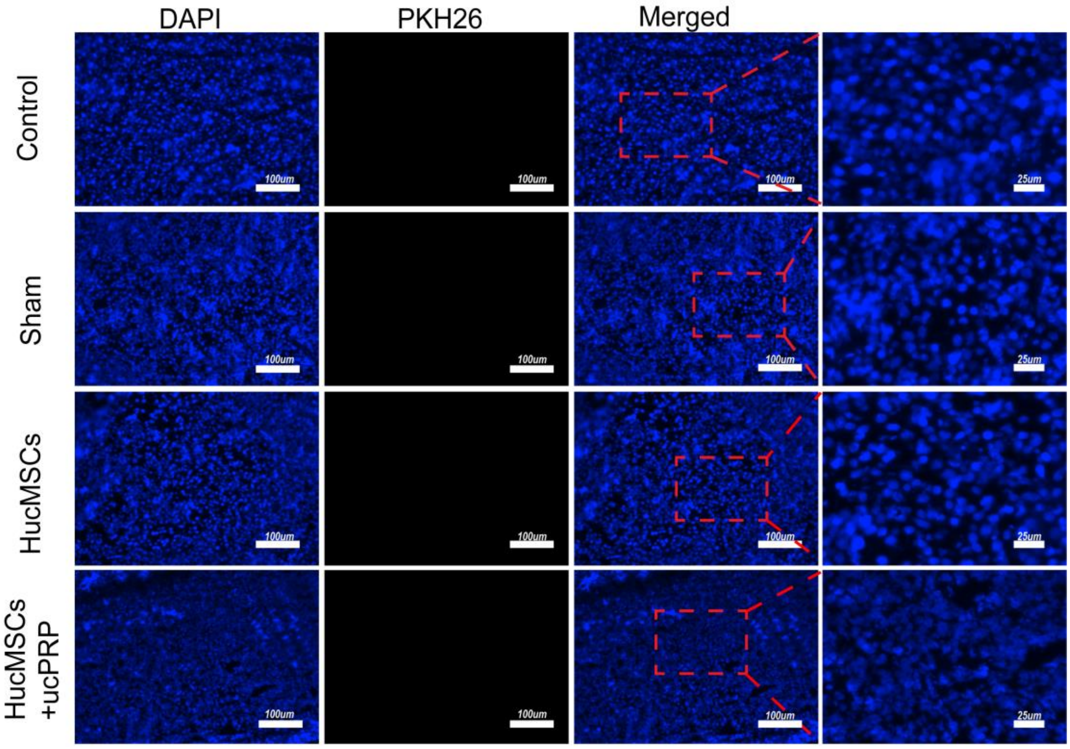
DAPI
PKH26
Merged
Control
Sham
HucMSCs
HucMSCs +ucPRP
100um
25um

J

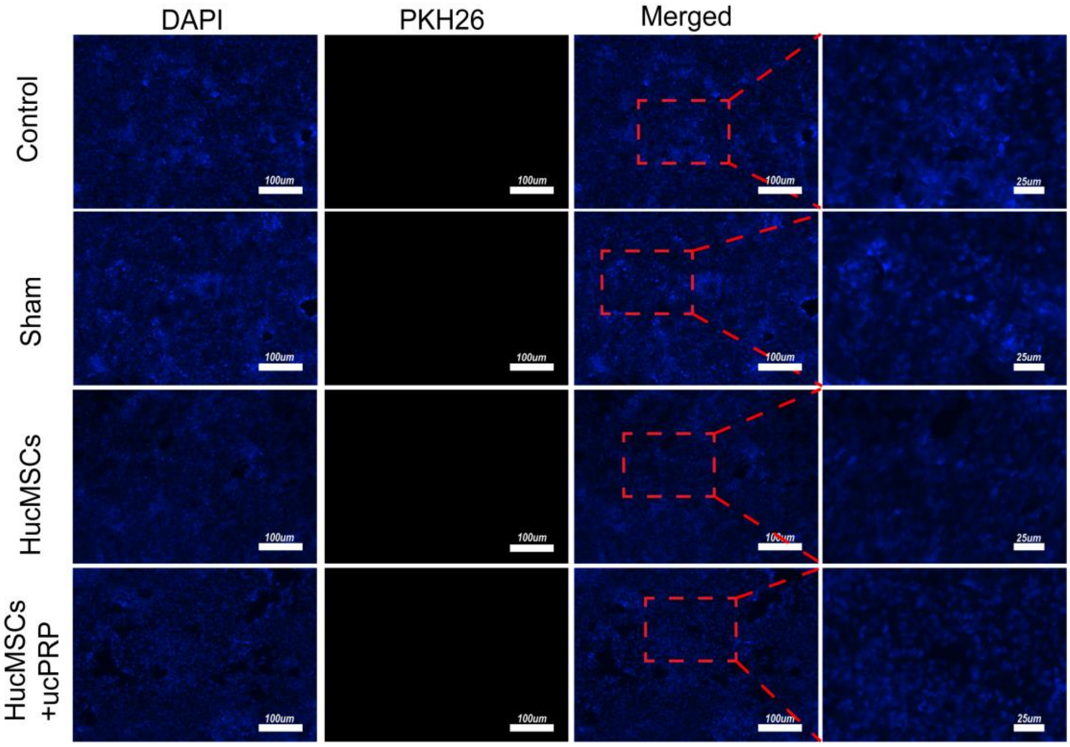
DAPI
PKH26
Merged
Control
Sham
HucMSCs
HucMSCs +ucPRP
100um
25um

K

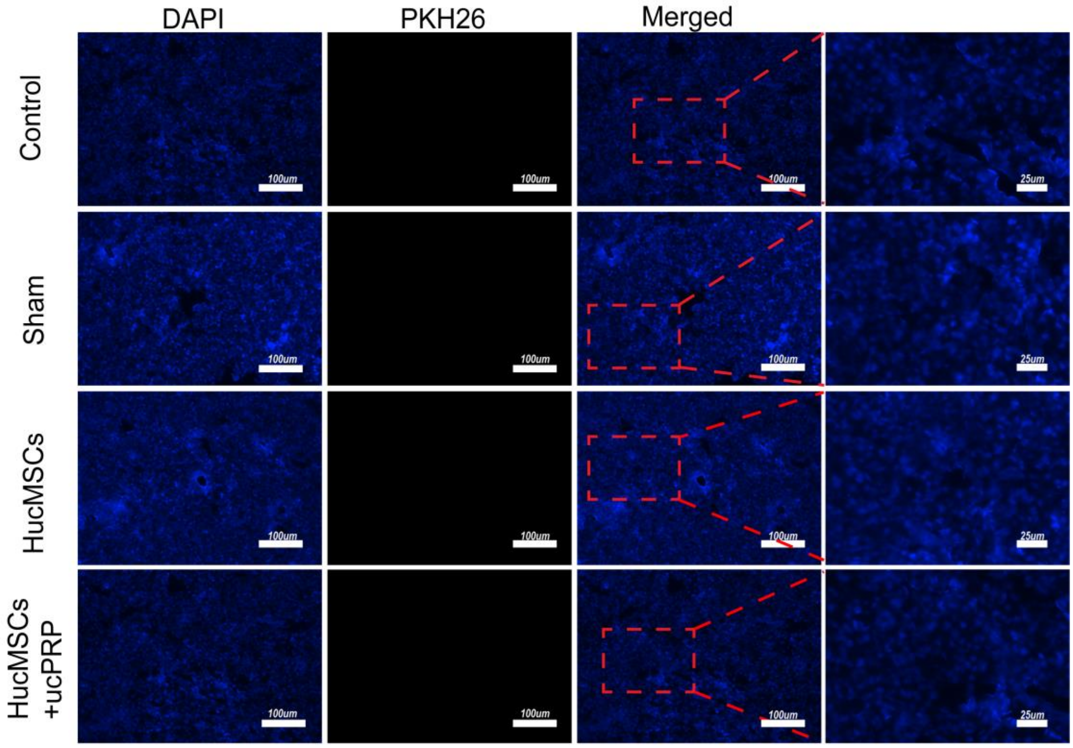
DAPI
PKH26
Merged
Control
Sham
HucMSCs
HucMSCs
+ucPRP
100um
25um

L

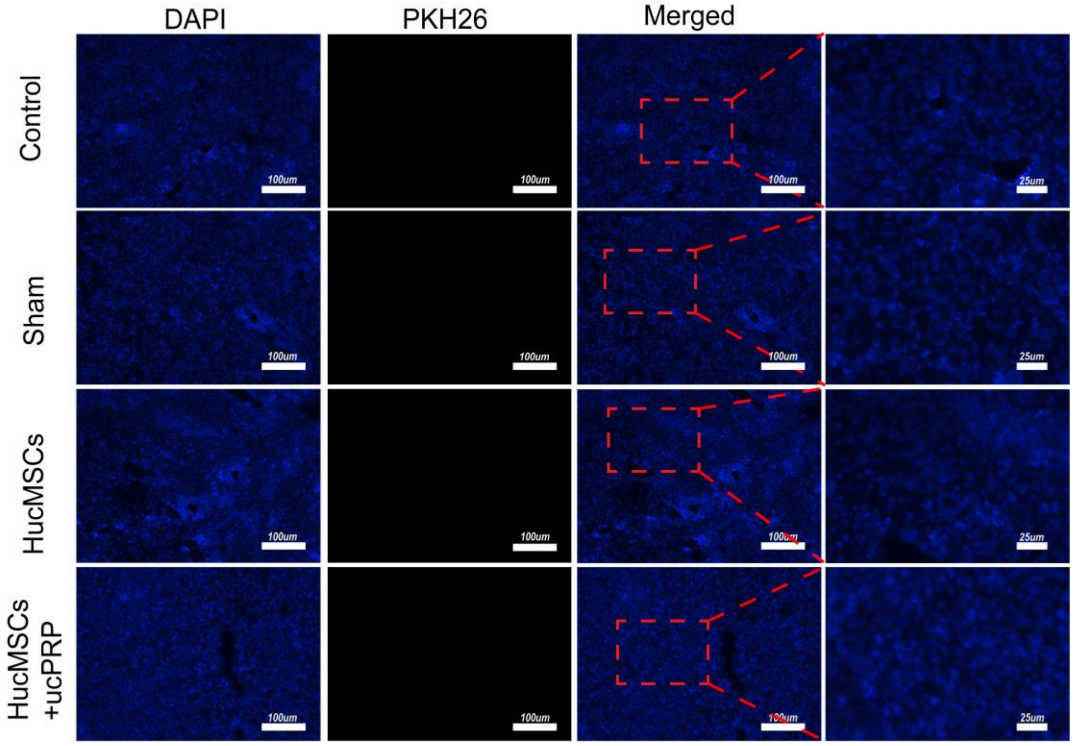
DAPI
PKH26
Merged
Control
Sham
HucMSCs
HucMSCs
+ucPRP
100um
25um

M

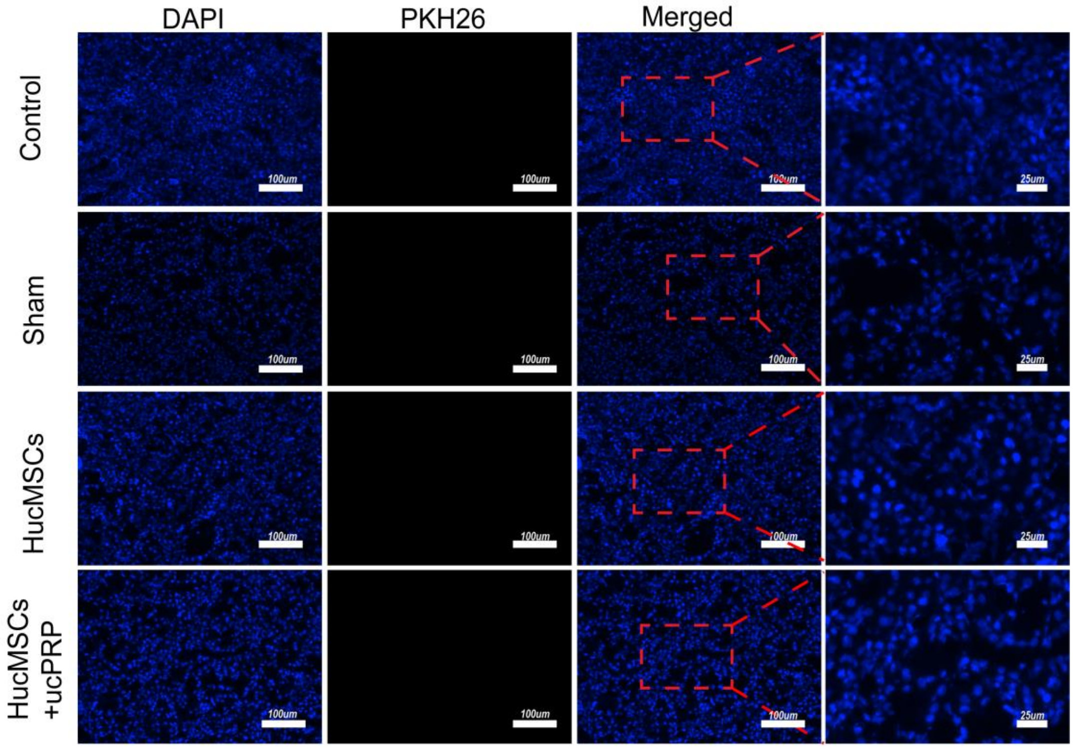
DAPI
PKH26
Merged
Control
Sham
HucMSCs
HucMSCs +ucPRP
100um
25um

N

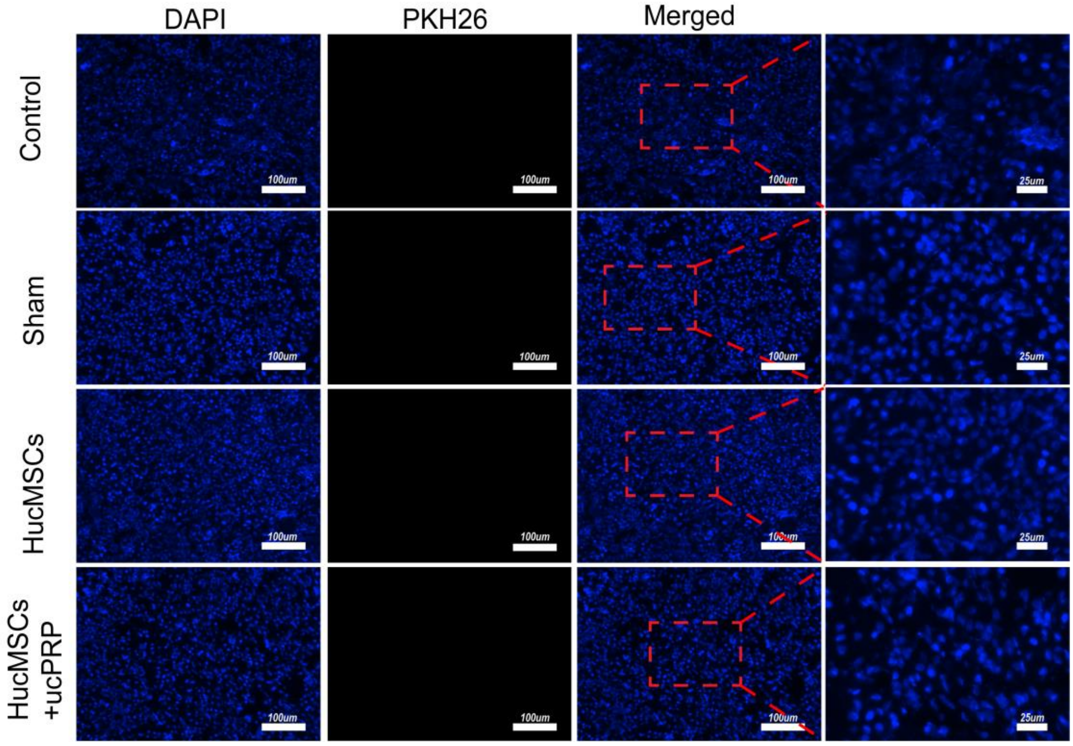
DAPI
PKH26
Merged
Control
Sham
HucMSCs
HucMSCs +ucPRP
100um
25um

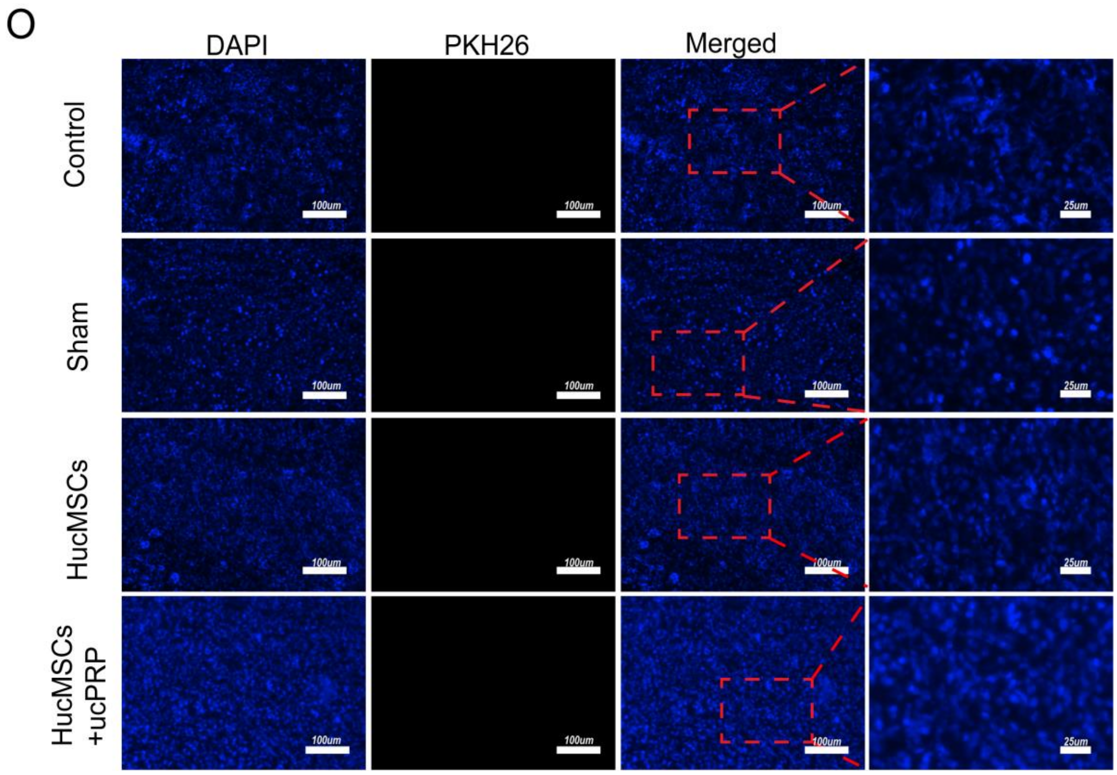

Figure S1: The distribution of HucMSCs in rat heart, liver, spleen, lung and kidney on D0, D15, and D30 after stem cells transplantation. (A) At D0, the distribution of HucMSCs in the heart. (B) At D15, the distribution of HucMSCs in the heart. (C) At D30, the distribution of HucMSCs in the heart. (D) At D0, the distribution of HucMSCs in the liver. (E) At D15, the distribution of HucMSCs in the liver. (F) At D30, the distribution of HucMSCs in the liver. (G) At D0, the distribution of HucMSCs in the spleen. (H) At D15, the distribution of HucMSCs in the spleen. (I) At D30, the distribution of HucMSCs in the spleen. (J) At D0, the distribution of HucMSCs in the lung. (K) At D15, the distribution of HucMSCs in the lung. (L) At D30, the distribution of HucMSCs in the lung. (M) At D0, the distribution of HucMSCs in the kidney. (N) At D15, the distribution of HucMSCs in the kidney. (O) At D30, the distribution of HucMSCs in the kidney.
